# Gastric mucinous adenocarcinoma presenting with chronic cough, bronchovascular bundle thickening, and multiple pulmonary nodules: a case report

**DOI:** 10.3389/fonc.2026.1713925

**Published:** 2026-01-28

**Authors:** Hongchun Huang, Fushou Chen, Hui Huang, Xiaofang Su, Minchao Duan

**Affiliations:** Department of Respiratory and Critical Care Medicine,Wuming Hospital of Guangxi Medical University, Nanning, Guangxi,, China

**Keywords:** bronchovascular bundle thickening, chronic cough, gastric mucinous adenocarcinoma, gastric signet ring cell carcinoma, pulmonary lymphangitic carcinomatosis

## Abstract

**Background:**

Chronic cough is a prevalent respiratory symptom, with etiologies spanning various systems. In the clinical setting, there may be an overlooking of the underlying gastrointestinal etiology in the context gastric cancer combined with pulmonary lymphangitic carcinomatosis presenting with chronic cough in the absence of gastrointestinal symptom. It may lead to missed diagnosis, posing a significant challenge to accurate diagnosis.

**Case presentation:**

A 52-year-old female patient was admitted to the hospital due to chronic cough lasting for five months. Chest CT revealed bronchial bundle thickening, interlobular septal thickening, and multiple small nodular opacities within the lungs. Gastroscopy indicated an ulcerative mass in the gastric body. Post-radical gastrectomy pathological examination revealed gastric mucinous adenocarcinoma, with approximately 20% exhibiting signet ring cell carcinoma. The diagnosis was gastric mucinous adenocarcinoma combined with pulmonary lymphangitic carcinomatosis. One month post-surgery, the patient commenced regular chemotherapy with the SOX regimen (Oxaliplatin 170mg d1+Tegafur-uracil 50mg,bid,d1-d14,q21d).The patient received 6 cycles of chemotherapy and experienced no recurrence through follow-up via gastroscopy. HRCT indicated an expansion in pulmonary lesions, but no extrapulmonary metastasis. However, one month after the discontinuation of intravenous chemotherapy, the patient was examined with multiple bone metastases in the sternum, scapulas, ribs, and thoracolumbar vertebral bodies, with expanded pulmonary lesions. Ultimately, the patient discontinued treatment due to respiratory distress.

**Conclusion:**

This study reports a case of gastric mucinous adenocarcinoma with pulmonary lymphangitic carcinomatosis presenting as chronic cough. Our case report highlights the importance of emphasizing chronic cough, encouraging a broader diagnostic approach, and actively seeking and confirming the existence of potential extrapulmonary diseases to prevent missed diagnoses and misdiagnoses.

## Introduction

Chronic cough typically manifests as sole or primary symptom, lasting for over 8 weeks, without significant detectable abnormality on chest X-ray examination. Its common etiologies generally include cough variant asthma, upper airway cough syndrome, eosinophilic bronchitis, allergic cough,gastroesophageal reflux-related cough, etc.

According to the definition of the World Health Organization, gastric mucinous adenocarcinoma is an adenocarcinoma presenting with a significant amount of extracellular mucoprotein within the tumor, constituting ≥50% of the tumor volume (1).As a rare subtype of gastric adenocarcinoma (2),it exhibits a high degree of invasiveness, along with a considerable rate of lymphatic metastasis.Typically,it is diagnosed at an advanced stage. In particular, compared with non-mucinous adenocarcinoma and signet ring cell carcinoma, gastric mucinous adenocarcinoma exhibits significantly higher post-treatment recurrence rate (3).Its poor prognosis may be indicated by the tumor diameter, lymph node metastasis, tumor stage, etc. (4).Noticeably, patients with advanced-stage disease experience a significantly poorer prognosis, although early-stage gastric mucinous adenocarcinoma does not differ in prognosis from other types of gastric cancer (2).

Furthermore, pulmonary lymphangitic carcinomatosis (PLC) is a specific type of lung metastatic cancer, manifesting as cancer cells metastasizing through the pulmonary lymphatic vessels. On imaging, its common features are interstitial abnormalities, such as interstitial thickening, nodular shadows, and bead-like patterns, with a high rate of misdiagnosis as interstitial lung diseases (5).The primary tumor types associated with PLC are predominantly adenocarcinomas, especially breast cancer, lung adenocarcinoma, gastric cancer, and colon cancer.

Lung metastatic cancer frequently manifests with symptoms characteristic of the primary disease. Here, we reported a case of gastric mucinous adenocarcinoma combine with PLC. The patient had a chronic cough, yet without pronounced symptoms of the primary disease.Upon initial diagnosis, the patient underwent high resolution computed tomography (HRCT),with the presence of bronchovascular bundle thickening in both lungs and significant interlobular septal thickening in the left lower lobe. These findings alter us to broaden the diagnostic approach for chronic cough to avoid missed diagnosis and misdiagnosis.

## Case presentation

A 52-year-old female, with recurrent coughing and expectoration persisting for five months, was admitted to the hospital on July 3,2024.The patient experienced paroxysmal dry cough, particularly severe at night during sleep. When the cough intensified, The patient reported accompanying symptoms of chest tightness and shortness of breath. However, she did not experience orthopnea, chest pain, hemoptysis. As for her medical history, the patient suffered from gallbladder stone and cholecystitis previously, with occasional pain in the middle-upper abdomen, but denied nausea, vomiting, hematemesis, or hematochezia.

**Figure 1:**
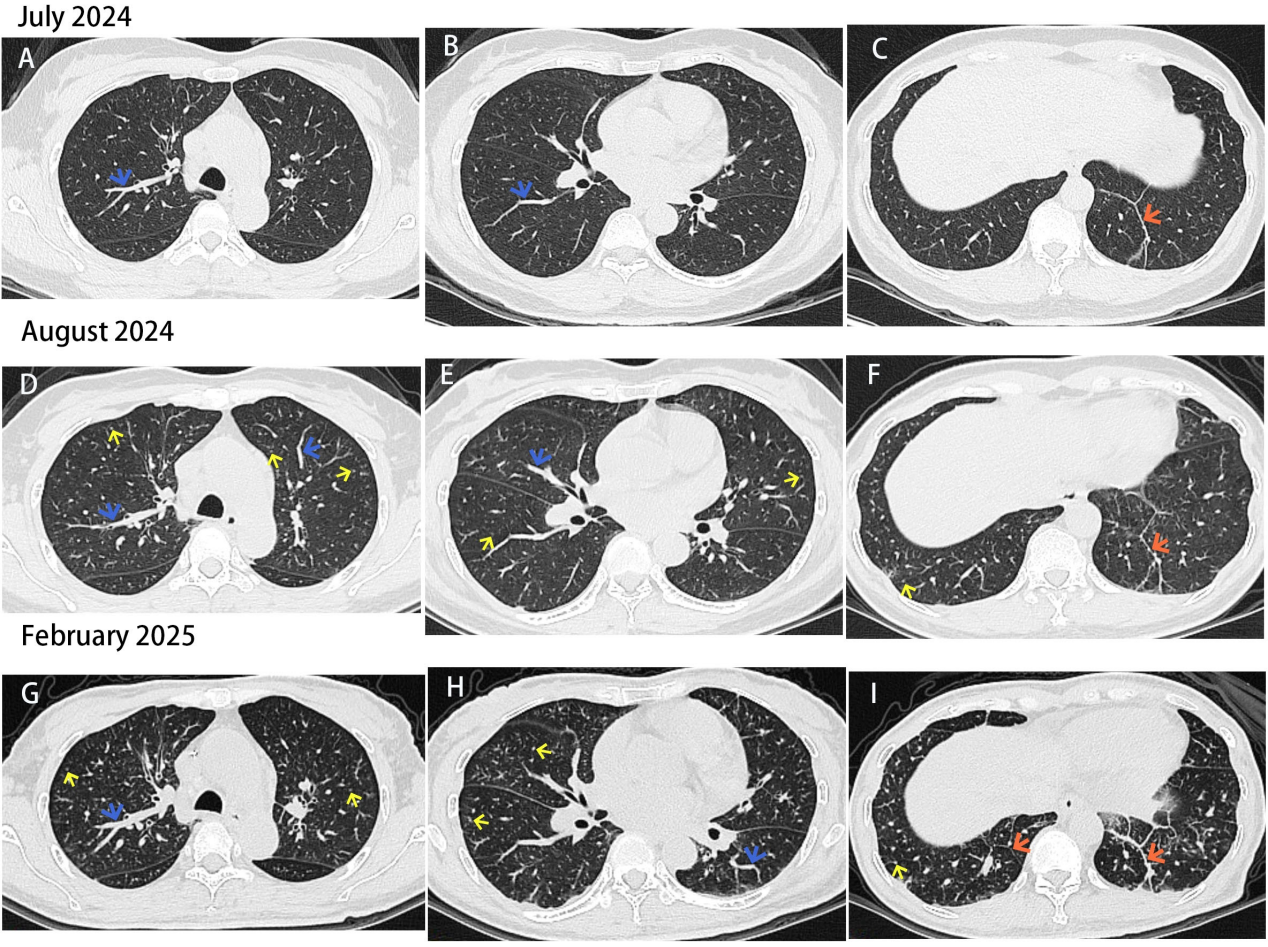
**(A, B, D, E, G, H)** Blue arrows indicate bronchovascular bundle thickening in both lungs. **(C, F, I)** Orange arrows demonstrate interlobular septa thickening in the lower lobes of both lungs. **(D, E, F, G, H, I)** Yellow arrows indicate multiple new small nodules in both lungs. All aforementioned lesions progress over time.

Laboratory examination:D-dimer:8.12 μg/mL (0.0-0.5 μg/mL).Serum total IgE:>500 IU/ml. Arterial blood gas analysis:pH:7.41,pCO_2_:36 mmHg,pO_2_:89 mmHg,PO_2_/FiO_2_:438 mmHg. Pulmonary function:VC:117.5%,FEV1:116.4%,FEV1/FVC:82.04%,indicating normal pulmonary ventilation function. Tumor markers:CEA:23.65 ng/mL (0.0-5.0 ng/mL),CA 72-4: 64.46 U/mL (0.0-6.9 U/mL).Cardiac ultrasound and CT scan of the paranasal sinuses: No abnormalities. CT scan of the upper abdomen: Gallbladder and common bile duct stone with cholecystitis to be confirmed, which was subsequently excluded by magnetic resonance cholangiopancreatography. Furthermore, a vascular ultrasound of lower limbs was performed for the patient, given her significantly elevated D-dimer level and the symptom of shortness of breath, which showed no evidence of embolism. Computed tomography pulmonary angiography (CTPA):1.No thrombus signs;2.thickening of bronchovascular bundles in the right upper, middle, and lower lobes, as well as the left lower lobe. Interlobular septa thickening was observed in the posterior basal segment of the left lower lobe (**Figures 1A-C**).The preliminary diagnosis was chronic cough. The patient was treated with inhalation of budesonide, ipratropium bromide, and acetylcysteine, as well as oral administration of loratadine and ketotifen. However, the patient had no improvement in cough symptom.

**Figure 2:**
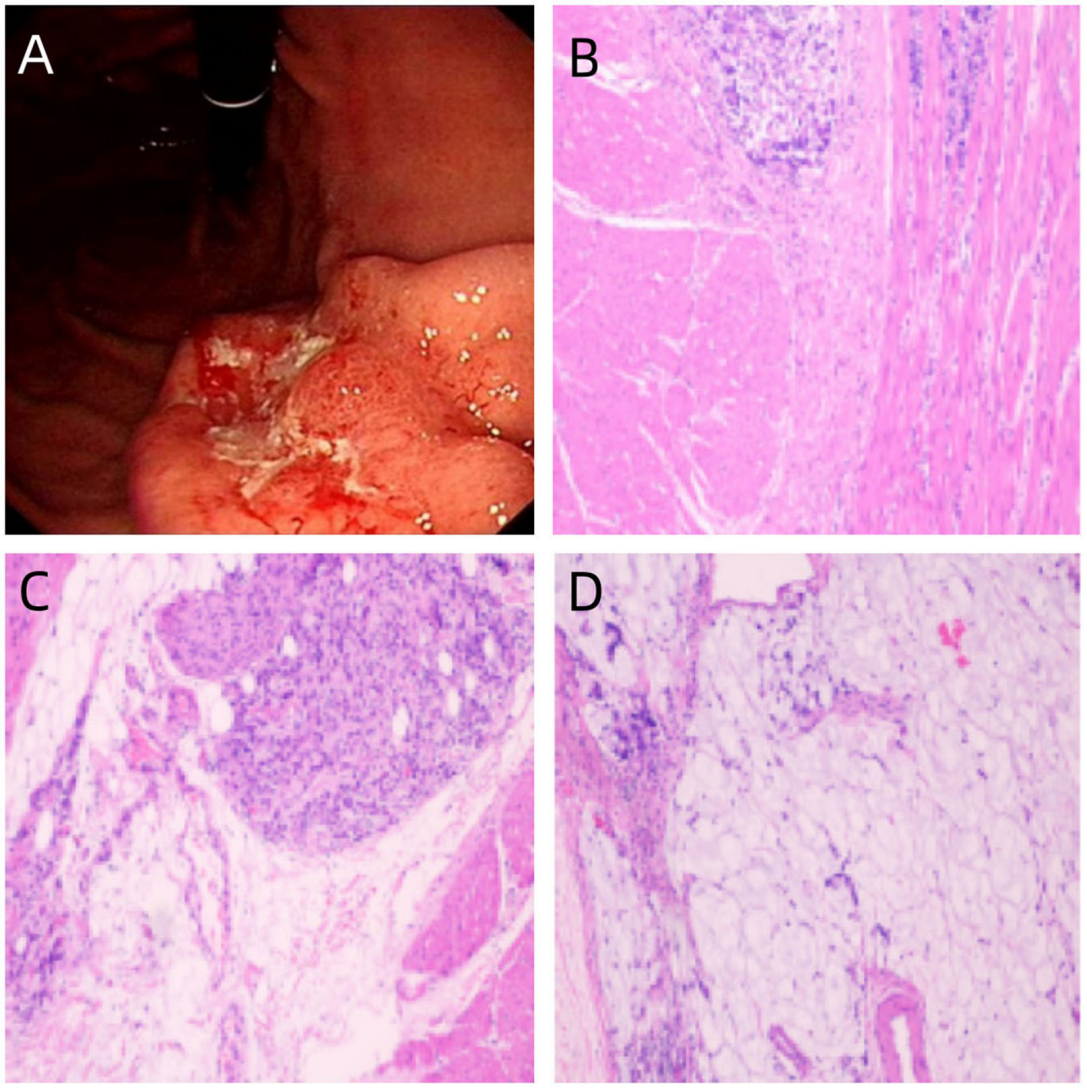
**(A)** Gastroscopy reveals an ulcerative mass in the gastric body. **(B–D)** Pathology reveals gastric mucinous adenocarcinoma with a component of signet-ring cell carcinoma.

**Figure 3:**
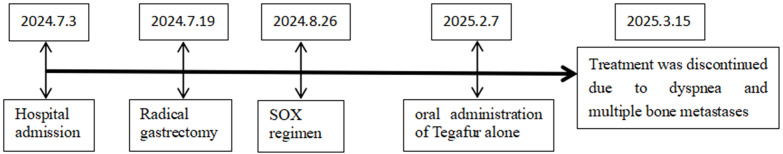
Timeline summarize the case diagnosis and treatment process.

The possibility of reflux esophagitis could not be excluded given that the patient experienced cough while lying down at night. Consequently, the patient was provided with pantoprazole and domperidone empirically for treatment, yet yielding no alleviation in coughing. Gastroscopy examination: A gastric body ulcer-like mass, with the nature to be determined (**Figure 2A**). The patient was transferred to the Department of Gastrointestinal Surgery.Whole-body CT scan:1.Localized edema and thickening of the gastric wall on the lesser curvature side of the stomach;2.no significant abnormality in the CT scan of the head, lower abdomen, and pelvis. On July 19,2024,the patient underwent a radical gastrectomy for gastric cancer. Postoperative pathological examination results indicated a gastric mucinous adenocarcinoma in the distal stomach, with approximately 20% of the tumor exhibiting characteristics of signet ring cell carcinoma. Moreover,the Lauren classification suggested mixed type; the tumor size was 4cm×2.5cm×1.2cm;and 19 lymph nodes revealed metastasis. Furthermore immunohistochemistry showed CK7 (+),CK20 (partially +),Villin (+),HER-2 (0,–),CKP (+),Ki67 (+,30%).CD34 (+),and S-100 (+) of the cancer cells, suggesting vascular cancer and nerve invasion. The detection of MLH1 (+),MSH2 (+),MSH6 (+),and PMS2 (+) implied intact mismatch repair function, with no protein loss (**Figures 2 B–D**).On August 26,2024,the patient was re-examined by enhanced CT scan, demonstrating the presence of mediastinal lymph nodes, along with multiple newly identified small nodules in both lungs. Additionally, there was thickening in the original bronchial vascular bundles and the interlobular septa lines of the left lower lobes of the lungs, consistent with previous findings (**Figures 1D–F**). The patient was diagnosed with gastric mucinous adenocarcinoma combined with PLC. On August 27,2024,the patient was prescribed with standardized chemotherapy with the initial SOX regimen (Oxaliplatin 170mg d1 + Tegafur-uracil 50mg,bid,d1-d14,q21d),with 6 cycles totally. We present the treatment timeline in **Figure 3**. On February 6,2025,the patient underwent a gastroscopy, displaying no recurrence of tumor. CT examination did not indicate any tumor recurrence or extrapulmonary metastatic lesions. However, compared to previous assessment, there was an increase in the bronchovascular bundles, interlobular septa lines thickening, and small nodulars in both lungs (**Figures 1G–I**). The patient was recommended to receive continuous treatment with local radiotherapy in conjunction with the SOX regimen. The patient refused both radiotherapy and intravenous oxaliplatin chemotherapy, and started oral administration of Tegafur at a dose of 40mg/m^2^ on February 7,2025.On March 15,2025,the patient was readmitted due to post-activity shortness of breath and lower back pain. CT enhancement scan revealed multiple bone metastases in the sternum, scapulas, ribs, and thoracolumbar vertebraes. CTPA showed no signs of embolism. The following day, the patient developed rapidly deteriorated condition, marked by a decrease in heart rate and blood pressure, leading to the abandonment of treatment.

## Discussion

Coughing is one of the most prevalent symptoms in the Respiratory Department. With a multifactorial etiologies, coughing may be induced by gastrointestinal disorder, cardiovascular condition,neurological disorder, and drug-induced cough, in addition to respiratory diseases. Psychological factor, such as conversion disorder, can also contribute to the manifestation of cough. In our report, the patient had no underlying medical history such as hypertension, coronary heart disease, or heart valve disease. Cardiac ultrasound showed no structural change, eliminating the possibility of cough caused by cardiovascular system diseases. The patient only presented with symptoms of cough and shortness of breath, without any related symptom of nerve damage, and no abnormality was found in neurological examination, thus excluding cough caused by neurological disorders. The patient also did not have cough resulted from drug use or conversion disorder given the absence of history of long-term medication use or neuropsychiatric symptoms. Chest CT indicated no abnormality in the pleura, excluding cough caused by pleural diseases. The localization of organic lesion is generally considered to be in the respiratory tract, and gastrointestinal diseases cannot be excluded due to the presence of abdominal pain. In this case, the patient had a major symptom of cough for 5 months. Initially, the chest CT showed only a few linear lesion, consistent with the definition of chronic cough.

In the diagnosis of chronic cough, it should be differentiated from that of common etiologies such as cough variant asthma, upper airway cough syndrome, eosinophilic bronchitis, allergic cough, and gastroesophageal reflux-related cough. The patient has no familial-hereditary disease of asthma, no history of allergic rhinitis, and no symptom of irritative cough. With normal results in pulmonary ventilation function test, the patient had no improvement in symptoms after bronchodilator inhalation, which did not support a diagnosis of cough variant asthma.T he upper airway cough syndrome as the cause of coughing was further excluded considering that the patient had no rhinitis symptom, and no abnormality in sinus CT scans. Although sputum cytology for eosinophils was not performed for the patient, the diagnosis of eosinophilic bronchitis was insufficiently supported given the absence of chronic irritating cough and no improvement with inhaled corticosteroid in this patient. Meanwhile, the possibility of allergic cough should be considered as the patient presented with chronic cough, accompanied by elevated serum total IgE. However, the patient did not have allergic disease or allergen exposure, and was non-responsive to glucocorticoids and antihistamine, making the diagnosis of allergic cough insufficiently supported. The possibility of gastroesophageal reflux disease remained to be excluded since the patient experienced an aggravated cough when lying flat and a history of abdominal pain. With a gastroscopy scheduled, the result, however, revealed smooth esophageal mucosa without congestion under endoscopy, and the patient showed no improvement with acid suppression, gastric protection, and prokinetic therapy, rendering the diagnosis of gastroesophageal reflux disease inadequately substantiated. In addition, the patient had no hypoxia via arterial blood gas analysis, without any direct or indirect signs of pulmonary embolism on CTPA, with pulmonary embolism ruled out eventually.

Noticeably, an ulcerative gastric mass was noticed during the gastroscopy examination to rule out gastroesophageal reflux disease. The pathology suggested gastric mucinous adenocarcinoma, with a component of signet ring cell carcinoma, both of which are highly malignant tumors with a high risk of lymphatic metastasis. Moreover, HRCT showed bronchovascular bundle thickening, interlobular septal thickening, and multiple small nodules in both lungs. Altogether, we speculated that the etiology of chronic cough in this patient might be attributed to the PLC caused by gastric mucinous adenocarcinoma.

As for PLC,the primary tumor is mostly adenocarcinoma, commonly involving breast cancer, lung adenocarcinoma, and gastric cancer (5, 6). Relevant patients may usually present with symptoms such as breathing difficulties and coughing, with higher possibilities of missed diagnoses and misdiagnoses. With the absence of specificity for its, PLC is typically diagnosed by pathological tissue biopsy, a gold standard for diagnosis currently. However, the presence of shortness of breath in patients may introduce significant risks associated with invasive biopsy procedures, posing considerable challenges to accurate diagnosis. According to report, only one case was successfully diagnosed among the 22 cases of PLC that underwent autopsy (7). The patient in this case was initially scheduled for a bronchoscopy, but did not undergo given the discovery of a tumor during gastroscopy. In general, clinical symptoms combined with imaging findings are referenced to confirm the diagnosis when factors such as dyspnea preclude bronchoscopic biopsy. HRCT demonstrates relatively characteristic lesions indicative of PLC. Additionally, the signs observed on HRCT that are beneficial for diagnosing PLC include smooth or nodular septa, subpleural nodules, bronchovascular bundle thickening, satellite nodules, and enlarged lymph nodes (8–11). Among these, bronchovascular bundle thickening and interlobular septa thickening are more prevalent manifestations in PLC (9, 11).

The application of PET/CT is instrumental in the diagnosis of PLC. In particular,18F-FDG PET/CT demonstrated specificity and sensitivity of over 84% and 86% in diagnosing PLC, respectively (12). HRCT and PET/CT exhibit comparable diagnostic performance in the detection of PLC (11). However,18F-FDG PET/CT exhibits very low detection rate for gastric mucinous adenocarcinoma and signet ring cell carcinoma, with the rate being negatively correlated with the amount of mucin protein within the tumor mass (13). In the pathological examination, the results were mucinous adenocarcinoma and signet ring cell carcinoma, both of which secrete mucin proteins. Given the low detection rate of PET/CT, we opted not to arrange for this examination. Here, this case was diagnosed with gastric mucinous adenocarcinoma with a signet ring cell carcinoma component, both of which exhibit a high degree of malignancy and a significant rate of lymphatic metastasis. The patient presented with chronic cough as the main clinical symptom, occasionally accompanied by wheezing. Imaging revealed bronchovascular bundle thickening, interlobular septal thickening, multiple small nodules in the lungs, and mediastinal lymphadenopathy, all of which indicated progressive progression. All these findings supported the clinical diagnosis of gastric mucinous adenocarcinoma in conjunction with PLC. So far, there is no report of gastric mucinous adenocarcinoma mixed with signet-ring cell carcinoma accompanied by PLC. Initially, the diagnosis of PLC was not considered in this patient, as signs such as bronchovascular bundle thickening and interlobular septal thickening are common in pulmonary inflammatory diseases. This diagnosis was consequently considered when progressive bronchovascular bundle thickening, interlobular septal thickening, and increase of pulmonary nodules were found in repeated chest CT scans. The patient presented with respiratory symptoms as the sole manifestation, without obvious gastrointestinal symptoms. The initial chest CT revealed only linear opacities, bronchovascular bundle thickening and interlobular septal thickening were occurred as the disease progressed. Although multiple small nodules were observed, their distribution pattern remained inconsistent with PLC, thus complicating diagnosis and increasing the risk of oversight. It imposes a challenge to diagnose PLC prior to cancer confirmation, particularly in the context of absent primary tumor symptoms while the presence of respiratory manifestations only, as it can easily be confused with respiratory diseases. During this stage, patients often present with dyspnea that may hinder the sampling of pathological specimens through invasive operations. Moreover, the obtained tissue samples may be inadequate when using fine-needle aspiration biopsy of small nodules. Critically, the accurate pathological diagnosis of small nodules, bronchovascular bundle thickening, and interlobular septal thickening depends on the harvest of larger tissue specimens. Such invasive procedures may also trigger significant risks of complications, such as pneumothorax and hemoptysis, further exacerbating dyspnea and even posing life-threatening. Therefore, it is still arduous to diagnose PLC by harvesting lung tissue for pathological biopsy, even when a primary tumor has been identified, which may limit our understanding of the disease. Consequently, signs such as bronchovascular bundle thickening and interlobular septal thickening should be highly concerned clinically as these may indicate underlying occult malignancy. Confirmation of the primary tumor serves as critical evidence for PLC diagnosis.

Gastric cancer is a prevalent primary tumor that frequently coexists with PLC. Their concurrent occurrence, typically insidious, often presents without digestive tract symptoms. At diagnosis, most patients have usually experienced distant metastasis (10, 14, 15).Due to the inherent capacity of the stomach for expansion, digestive tract symptoms are seldom observed in the involved patients at the early stage of cancer. This patient occasionally experienced abdominal pain, which was mistakenly attributed to a recurrence of cholecystitis, leading her to dismiss its significance. The patient showed respiratory symptoms, including a chronic cough and occasional shortness of breath, resembling previously reported cases of gastric cancer associated with PLC (6, 16).As documented previously, the most common types of gastric cancer include gastric signet ring cell carcinoma (16, 17) and poorly differentiated adenocarcinoma (18).As a highly invasive tumor (19),gastric mucinous adenocarcinoma has a high rate of lymph node metastasis, with a frequent diagnosis at an advanced stage in most cases (3).In our case, the histopathological composition was gastric mucinous adenocarcinoma predominantly, with 20% being gastric signet ring cell carcinoma. Commonly, both types of tumors described before secrete mucin proteins, with the former having mucin located extracellularly and the latter intracellularly. It has been recognized that mucin proteins, encoded by MUC (20),are involved in the initiation and progression of tumors.MUC2,with a high expression in mucinous adenocarcinoma, serves as a reliable marker for mucinous cancer (21).The expression of MUC2 exhibits an intimate association with the depth of invasion and lymph node metastasis (22).Moreover, high mucin content and elevated MUC2 expression might also related to a poorer survival in the affected patients (23).Critically, gastric mixed-type signet-ring cell carcinoma, characterized by a proportion of signet-ring cell carcinoma of <50%,demonstrates a higher rate of lymph node metastasis compared to conventional gastric signet-ring cell carcinoma, where the proportion exceeds 50% (24, 25).Mixed adenocarcinoma is more invasive, which may result in a poorer prognosis (24).It may partly explain the early development of PLC in the patient. There are three pathways,i.e., hematogenous, pleural, and lymphatic metastasis, for pulmonary metastasis of gastric cancer (26).Gastric mucinous adenocarcinoma is particularly prone to abdominal implantation (4).However, in this case, we noticed no other common metastatic sites, such as abdominal implantation, aside from the primary tumor, surrounding lymph nodes, mediastinal lymph nodes, and PLC. We speculated that the tumor metastasized to the lungs via the mediastinal lymph nodes, given the absence of multiple nodular masses in the pulmonary imaging and the lack of pleural effusion in this case.

Currently, treatment of the primary tumor remains the major concern and there is still no effective treatment for PLC. In the early stage, it is commonly to adopt a comprehensive treatment plan targeting the primary tumor is chosen, while there is limited efficacy when applying treatments for respiratory symptoms(e.g., antitussive, antispasmodic, and even hormone).Surgery and chemotherapy are effective for treating gastric mucinous adenocarcinoma. Critically, concerning the molecular mechanism of lymphatic metastasis in malignant tumors, the spread of pulmonary metastatic tumors within the lymphatic system may be promoted given that VEGF-C can induce dilation of pulmonary lymphatic vessels and promote the survival of metastatic lymphatic vessels (27).Therefore, as reported previously, VEGF-C-targeting therapy can improve the survival and prognosis of PLC. Apatinib, which targets VEGF, has demonstrated potential benefits. For instance, in a case report, low-dose apatinib, in combination with comprehensive tumor treatment, can improve patient survival (28).Similarly, using Bevacizumab to target VEGF can also benefit the prolongation of PLC patients’ survival significantly (29).

In this case, the patient underwent a radical gastrectomy for gastric cancer, based on the established guidelines, and given regular SOX regimen post-operatively. No evidence of tumor recurrence or metastasis beyond the lungs was observed in this patient throughout the six cycles of chemotherapy after diagnosis, indicating the effectiveness of the treatment plan. However, the prognosis of PLC is exceedingly poor (30–33),with a reported mortality rate of approximately 50% within three months after the onset of respiratory symptoms (34).In this case, the patient survived for 14 months following the onset of respiratory symptoms, and discontinued treatment for dyspnea and multiple bone metastases 8 months after diagnosis.

In summary, we report a case of gastric mucinous adenocarcinoma combined with PLC presenting as chronic cough. The pathological component is a mixed type of gastric mucinous adenocarcinoma, with signet ring cell carcinoma accounting for 20%.The patient was imaged with bronchovascular bundle thickening in both lungs, accompanied by multiple small nodules. Unfortunately, we were unable to obtain lung tissue pathology for a definitive diagnosis. This case serves as a critical reminder that chronic cough should be highly valued as it may also indicate other systemic conditions, beyond respiratory diseases. Therefore, it is imperative to expand our diagnostic strategies to facilitate early detection of underlying diseases, and to prevent both misdiagnosis and missed diagnoses in the clinical setting.

## Data Availability

The raw data supporting the conclusions of this article will be made available by the authors, without undue reservation.
